# Multiscale positive feedbacks contribute to unidirectional gastric disease progression induced by helicobacter pylori infection

**DOI:** 10.1186/s12918-017-0497-y

**Published:** 2017-11-22

**Authors:** Richard Ballweg, Frederick Schozer, Kelsey Elliott, Alexander Kuhn, Logan Spotts, Eitaro Aihara, Tongli Zhang

**Affiliations:** 1 Department of Molecular and Cellular Physiology, College of Medicine, University of Cincinnati, Cincinnati, OH USA; 2 Division of Plastic Surgery, Department of Surgery, Cincinnati Children’s Hospital Medical Center, Cincinnati, OH USA; 3 Division of Developmental Biology, Department of Pediatrics, Cincinnati Children’s Hospital Medical Center, Cincinnati, OH USA

## Abstract

**Background:**

Helicobacter Pylori (HP) is the most common risk factor for gastric cancer. Nearly half the world’s population is infected with HP, but only a small percentage of those develop significant pathology. The bacteria itself does not directly cause cancer; rather it promotes an environment that is conducive to tumor formation. Upon infection, HP induces transcriptional changes in the host, leading to enhanced proliferation and host immune response. In addition, HP causes direct damage to gastric epithelial cells.

**Results:**

We present a multiscale mechanistic model of HP induced changes. The model includes four modules representing the host transcriptional changes in response to infection, gastric atrophy, the Hedgehog pathway response, and the restriction point that controls cell cycle. This model was able to recapture a number of literature reported observations and was used as an *“*in silico*”* representation of the biological system for further analysis. Dynamical analysis of the model revealed that HP might induce the activation of multiple interplayed positive feedbacks, which in turn might result in a “*ratchet ladder*” system that promotes a unidirectional progression of gastric disease.

**Conclusions:**

The current multiscale model is able to recapitulate the observed experimental features of HP host interactions and provides dynamic insights on the epidemiologically observed heterogeneity in disease progression. This model provides a solid framework that can be further expanded and validated to include additional experimental evidence, to understand the complex multi-pathway interactions characterizing HP infection, and to design novel treatment protocols for HP induced diseases.

**Electronic supplementary material:**

The online version of this article (10.1186/s12918-017-0497-y) contains supplementary material, which is available to authorized users.

## Background

The most common risk factor for developing gastric cancer is the stomach pathogen, Helicobacter pylori (HP), which infects half of the world’s population [1]. Normally benign, HP has the capacity to cause damage to the host’s gastric mucosa by inducing a chronic inflammatory response, a necessary step towards more serious pathologies.

Humans are the primary reservoir for HP, which is thought to pass between individuals though fecal-oral or oral-oral routes of infection [2]. After entering the mouth, the gram-negative, rod shaped bacteria enter the stomach and adhere to columnar epithelial cells, primarily in the corpus and antrum due to their relatively low pH environments [3, 4]. Once secure, the bacteria secrete urease which converts urea, naturally occurring in the stomach, to bicarbonate and ammonia [3, 4]. These compounds increase the pH environment of the stomach allowing HP to thrive.

The combined effects of the host’s immune response and HP virulence factors can induce chronic gastritis in previously normal gastric mucosa. Over time, the stress caused by chronic inflammation results in full gastric atrophy, which is a significant development towards gastric cancer [5].

Studies have shown that HP gene products induce host transcriptional regulatory factors, such as NF-κB, to produce cytokines and compel the host to mount an immune response [6]. In an otherwise healthy stomach, HP colonization of the gastric epithelium leads to the recruitment of monocytes that secrete inflammatory cytokines (e.g. IL-12, TNF-alpha, IL-1Beta, IL-6 and IL-8) [7]. Chronic inflammation of the stomach by cytokines causes significant damage to existing glandular epithelial cells, prompting affected cells to undergo apoptosis [3].

Compounding its inflammatory effects, HP produces virulence factors that further damage the gastric epithelium and aid it in avoiding the host’s immune response. The outer membrane proteins of HP (e.g. BabA and OipA) cause cellular toxic responses through direct binding to the membrane proteins outside of epithelial cells. In addition, HP secretes VacA proteins into the outer cellular space and inject CagA proteins directly into epithelial cells. Without antibiotics, the infected host is often incapable of stopping this damage, as HP successfully evades the efforts of the host immune response [3, 8].

Multiple mathematical models have been constructed to investigate the dynamical properties of the molecular and cellular responses to HP. However as elaborated in the discussion, there still lacks a comprehensive mechanistic framework for understanding and predicting disease progression upon HP infection. In particular, two essential questions remain to be answered:

1. Pathogenesis: how does infection by HP promote the pathological progression of disease?
2. Heterogeneity: why, among all the people infected by HP, do some readily develop severe disease while others do not?

In this work, we have constructed a mechanistic model to describe the host pathogen interactions during HP infection and the resulting immune response. The current model incorporates many essential host responses to HP including: the transcriptional changes in response to the pathogen, gastric atrophy, and cell proliferation.

Dynamical analysis of the model predicts that HP infection disrupts a chain of molecular switches formed by positive feedbacks. Importantly, due to the dynamical nature of these predicted switches, disruption may contribute to unidirectional disease progression, even if the signals induced by the pathogen are transient. If true, this suggests that gastric pathology might persist even in the absence of HP, and that removal of the pathogen may not be sufficient to treat or reverse the damage already done by the bacteria.

Our hope is that by further understanding the mechanisms involved in HP induced gastric disease, the scientific community might develop more advanced prevention methods to either prevent infection or the damage caused by the pathogen. Prevention is vitally important, given that the disease is often discovered in its advances stages. Another important goal is to develop therapies that target vital mechanisms of the pathogenic pathway and possibly target pathways that could prevent a normally benign HP infection from developing into more significant pathology.

## Results

### The cellular and molecular interactions in the current model

**Fig. 1:**
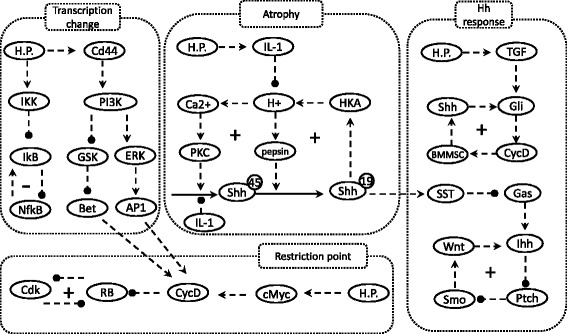
The model wiring diagram. Nodes represent the model components, arrows indicate activation, and circle heads indicate repression. The symbols “+” and “-” indicate positive and negative feedbacks, respectively. The model consists of four functional modules, which are separated into different rectangles for easier visualization. The molecular interactions of these modules are elaborated in the Method section

To understand the complex cellular and molecular responses to HP infection, we have summarized the currently prevailing hypotheses regarding HP infection and host responses into a wiring diagram. As a visualization aid, the wiring diagram is further divided into four modules (Fig. 1): the transcription changes, atrophy, Hedgehog singling, and the restriction point. The biological justifications for these modules are described in the method section.

Similar to experimental models, a mathematical model must be able to mimic the biological systems we wish to study. To fulfill this requirement, we first test whether our model is able to recapture experimental observations.

### Modeling host transcriptional changes following HP infection

Under normal conditions, NF-κB is held in its inactive state by its inhibitor IkB proteins, with IkBα being the dominant one. Upon infection with HP, gastric cells lose their IkBα expression within 2 h. This loss results in an increase in Nf-kB expression and a corresponding increase in downstream genes [6].

**Fig. 2:**
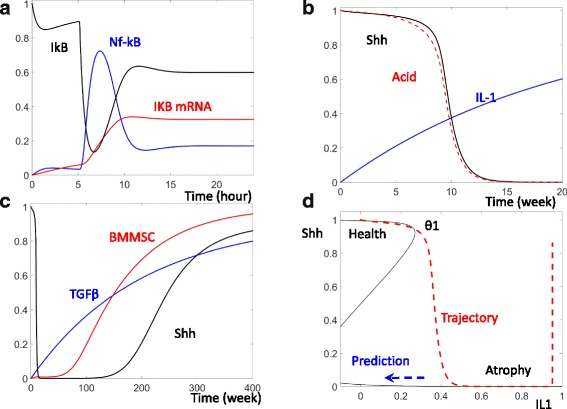
Dynamic change of Nf-κB and Shh following HP infection. **a** Temporal simulations of IkB proteins (black curve), Nf-κB(blue), and Nf-κB target genes (red) after induction of HP (at *T* = 5 h). **b** Temporal simulations of stomach acid (red), Sonic Hedgehog (Shh, black), and Interleukin-1 family of cytokines (IL-1, blue) after HP infection. **c** Temporal simulations of transforming growth factor beta (TGF-B, blue), BMMSC (red), and Shh (black) following HP infection. **d** The black curve shows the steady states of Shh as IL-1 level changes. TGF-β is set to be zero in this computation. The red curve shows the temporal changes of IL-1 and Shh from (**b**)

IkBα itself is a target gene of NF-κB, after its activation, Nf-κB promotes the expression of IkBα. This negative feedback has been incorporated into our current model (Fig. 1). Prior to HP infection, the model simulation shows a high level of IkBα (black curve, Fig. 2a) and a low level of NF-κB (blue curve, Fig. 2a). Following infection by HP (applied at *t* = 5 h in Fig. 2a), IkBα is degraded and its level quickly decreases. Due to the degradation of its inhibitors, NF-κB is activated. Activated NF-kB leads to the transcription of target genes (red curve, Fig. 2a). Because IkBα is downstream of Nf-κb, IkBα level gradually increases and again brings down the level of NF-κB.

Despite the function of the negative feedback, NF-κB activity is sustained for some time following HP infection. It was previously reported that NF-κB transcription activity can still be observed 24 h after HP infection [6]. Our model simulation is consistent with this experimentally observed activation of NF-κB transcription activity even in the presence of the re-accumulated IkBα (see Fig. 4A and B, Schumacher et al., 2015, [6]). The residual activity of NF-kB is able to sustain the transcription of its target genes for more than 20 h post HP infection (Fig. 2a).

In addition to NF-κB, HP infection also activates other transcriptional enhancers and factors such as β-catenin and AP1. In turn, these factors lead to increased CyclinD production and enhanced cell proliferation [9]. Such enhanced proliferation will be discussed below.

### The current model recaptures HP induced gastric atrophy

In the weeks following HP infection, elevated expression of IL-1 can be detected in infected mice [10]. This gradual increase in IL-1 is recaptured in our model simulation (blue curve, Fig. 2b). The increase in IL-1 is able to repress the secretion of acid as well as the production of Shh (see Fig. 5A and B, Waghray et al. 2009) [10]. After the level of IL-1 increases to a threshold point, both acid secretion and Shh expression are lost (Fig. 2b), consistent to the experimentally observed atrophy.

As illustrated in the wiring diagram (Fig. 1), a positive feedback between acid secretion and Shh production maintains high levels of both acid and Shh in healthy cells. After the increase of IL-1, Shh is inhibited and this positive feedback is blocked, leading to an atrophic state where upon gastric cells lose production of both Shh and acid (Fig. 2b).

### Shh reemerges in the presence of high IL-1

During chronic HP infection, elevated levels of inflammatory cytokines (e.g. INF-γ, TGF-β) recruit bone marrow derived mesenchymal stem cells (BMMSCs) to the site of gastritis [11, 12]. These BMMSCs express and secrete high levels of Shh [13]. This forms an autocrine loop in which Shh promotes the expression of Cyclin D1 in MSCs to promote MSC proliferation. Cyclin D1 represses the cell cycle inhibitors Rb and p27, leading to an increase in the percentage of MSCs in the S/G2/M stages. Through a paracrine loop, Shh also promotes the proliferation of epithelial cells, including CD44 positive tumor stem cells [14].

**Fig. 3:**
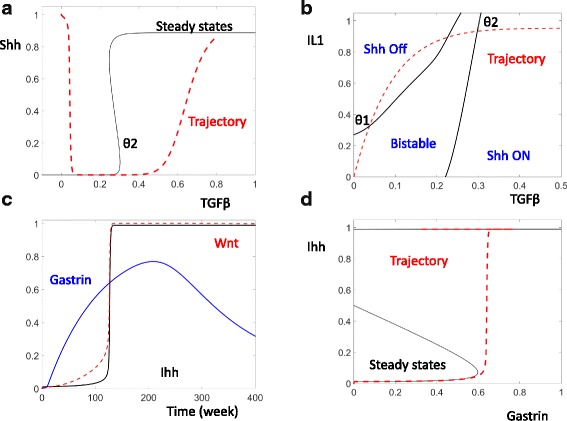
The dynamic control of Shh and Ihh. **a** The black curve shows the steady state of Shh for different levels of TGF-β. The level of IL-1 is set high (IL-1 = 1) to mimic a cell in the late stage of HP infection. The red curve shows the temporal change of TGF-β and Shh from Fig. 2c. **b** In response to the changes of both TGF-β and IL-1, the thresholds divide the TGF- β and IL-1 plane into three regions with inactivated Shh, activated Shh, and bistability. **c** Temporal simulations of Gastrin (blue), Wnt (red), and Indian Hedgehog (Ihh, black) following HP infection. **d** The black curve shows the steady state of Ihh as a function of the level of Gastrin. The red trajectory records the temporal changes of Ihh and Gastrin from (**c**)

This reemergence of Shh is mimicked in our model. As shown by the black curve in Fig. 2c, Shh level is decreased 10 weeks post HP infection. Later, the increased level of TGFβ (blue curve, Fig. 2c) recruits BMMSCs to the site of infection (red curve, Fig. 2c), (see Fig. 1, Houghton et al. 2004, [11] and Fig. 3, Varon et al. 2012, [12]). In turn, these MSCs can raise the Shh level months after HP infection.

### Gastric atrophy is likely controlled by a bi-stable Shh switch

Under normal physiological conditions, acid secreted by parietal cells along with Shh forms a positive feed-forward loop as shown in Fig. 1. Shh promotes the expression of proton pumps in parietal cells, thus increasing the secretion of acid. In return, the H+ secreted by parietal cells promotes the processing and activation of Shh. This mutual activation between Shh and acid sustains a healthy environment with both a high level of acid and Shh.

Following HP infection, the accumulation of inflammatory factors such as IL-1 results in the reduction of both Shh and acid (Fig. 2b). To better understand the time dependent change of Shh and its control by IL-1, we have computed the steady state of Shh at varying IL-1 levels (black curve, Fig. 2d). The time dependent change of Shh and IL-1 are also plotted (red curve, Fig. 2d).

In healthy cells, IL-1 level is low and Shh level remains high. Following HP infection, IL-1 begins to accumulate. Before IL-1 reaches the critical threshold (θ1, Fig. 2d), the high Shh level is maintained. After the level of IL-1 accumulates higher than the critical threshold, the steady state with high Shh level collapses with the intermediate, unstable, steady state and is lost. Following the loss of the high Shh state, Shh is attracted by the remaining steady state with low Shh. This remaining state may correspond to gastric atrophy.

If the positive feedback indeed functions as described, our model predicts that a bistable switch controls the conversion of the healthy state to the atrophy state. Furthermore, this bistability predicts that a lower level of IL-1 is necessary to maintain the atrophy state once atrophy is induced. In the extreme case indicated by the blue dashed arrow in Fig. 2d, the atrophy state might be maintained even after IL-1 disappears. In this way, the model predicts that a transient IL-1 increase might be sufficient to cause permanent gastric atrophy in infected patients.

### Shh reemergence is controlled by additional positive feedback

The upward movement of the red trajectory at high levels of IL-1 cannot be explained by the computed steady state of Shh (Fig. 2d). Rather, this late activation of Shh is caused by the accumulation of TGF-β, triggering an additional positive feedback (Fig. 3a). This feedback defines a second threshold for TGF-β (θ2, Fig. 3a). After TGF-β accumulates higher than this second threshold, the stable attractor with low Shh disappears and Shh level rises. The increase of Shh promotes the recruitment of BMMSCs, which secrete additional Shh. The mutual activation between Shh and BMMSC results in the reemergence of a high Shh steady state at high level of TGF-β. Note that the elevation of Shh and BMMSC are quite slow as shown by the red, time dependent trajectory. These components do not begin to increase until the level of TGF-β has passed over θ2 for quite some time (Fig. 3a).

### The interplay between the positive feedbacks that control Shh

The level of Shh is controlled by two independent elements IL-1 and TGF-β.The combined effect of these two control elements can be illustrated on the plane of IL-1 and TGF-β. In Fig. 3b, the thresholds of Shh activation and inactivation (black curves) divide the IL-1-TGF-β plane into three distinct areas: the left area is characterized by inactive Shh; the right area by activated Shh; in the middle bistable area, Shh could be either low or high depending on the system’s history.

The red, time dependent trajectory in Fig. 3b illustrates the comprehensive trajectory of Shh. In healthy cells, Shh level is active due to the positive feedback between Shh and acid secretion. After HP infection, the accumulation of IL-1 brings the system across the Shh inactivation threshold and results in a low level of Shh. Later, the accumulation of TGF-β brings the system across the Shh activation threshold and Shh accumulates once again. Compared with the isolated views that only considers IL-1 (Fig. 2d) or TGF-β (Fig. 3a), the IL-1 and TGF-β plane (Fig. 3b) might provide a satisfactory explanation for why Shh first decreases and then increases.

### The current model mimics the observed activation of Ihh

During the atrophy stages following HP infection, parietal cells are lost and Shh is repressed. This condition is mimicked in a parietal cell specific Shh knockout mouse. In these mice, reduction in Shh expression and acid production results in a decrease in somatostatin (SST) production by D cells; the reduced SST levels lead to elevated gastrin production (by G cells) and plasma gastrin. Circulating gastrin binds to gastrin receptors on gastric Pit cells, resulting in the expression of Ihh in these cells. After its production and secretion, Ihh binds to its trans-membrane receptor Pth located on mesenchyme cells, activating the downstream transcription factor Gli (in mesenchyme cells). Gli, in turn, elevates the expression of its transcription targets (i.e. Wnt). After Wnt is produced and secreted (in mesenchyme cells), it binds to receptors and stabilizes β-catenin in epithelial cells. Stabilized β-catenin results in elevated proliferation of epithelial cells [15].

The activation of Ihh is recaptured in our model simulation (black curve, Fig. 3c). The elevation of Ihh follows the elevation of the Gli target gene Wnt after the positive feedback Ihh is fully activated (red curve, Fig. 3c) (see Figs. 3, 5, 6 of Feng et al. 2014, [15]).

As described above, the elevation of TGF-β could cause an increase of Shh months after the initial the HP infection. Because decreased Shh might be responsible for the increase in gastrin, the reemergence of Shh should cause a decrease in gastrin, as simulated with the blue curve in Fig. 3c.

Despite the transient increase of gastrin, our model simulation suggests that the elevated levels of both Ihh and Wnt might be sustained (red and black curves, Fig. 3c). This is due to the activation of a positive feedback between Pit cells and Ihh. Pit cells secrete an elevated level of Ihh, while increased Ihh promotes the proliferation of more Pit cells. Due to this mutual activation, elevated Ihh levels can be sustained even after gastrin level decreases (the red trajectory, Fig. 3d). A model of this multi-scale feedback was elegantly illustrated by Feng et al. [15].

### The current model mimics HP induced proliferation

In the stomach, Lgr5+ stem cells reside at the base of antral glands. This stem cell pool continuously self-renews and differentiates into other functional cells. After HP enters the stomach, the bacteria colonizes the gastric glands resulting in enhanced proliferation of gastric stem cells.

Lgr5-eGFPIRES-CreERT2 and Rosa26-TdTomato mouse lines were crossed. Upon treatment with tamoxifen, the Lgr5+ cells and their progenitors expressed TdTomato [16]. After uninfected, control cells are treated with tamoxifen for 5 days, only a small portion of the antral gland is TdTomato+. At this low rate of proliferation, it took 10–15 days for the TdTomato + cells to replace the antral gland. In contrast, mice infected with HP show an increase in the accumulation rate of TdTomato + cells. Moreover, this enhanced proliferation correlates with the HP number in the antral gland, indicating that HP infection enhances the proliferation of Lgr5+ stem cells (i.e. the more bacteria, the stronger the proliferation) [16]. This increase in proliferation can be detected as soon as 24 h post infection [17].

Mammalian cells make the decision on whether or not to enter the cell cycle at their restriction point [18, 19]. At this point in the G1 phase of the cell cycle, the Rb activity in a cell is either lost or sustained high. In cells with sustained Rb activity proliferation is arrested. Conversely, cells with inactive Rb enter active proliferation.

**Fig. 4:**
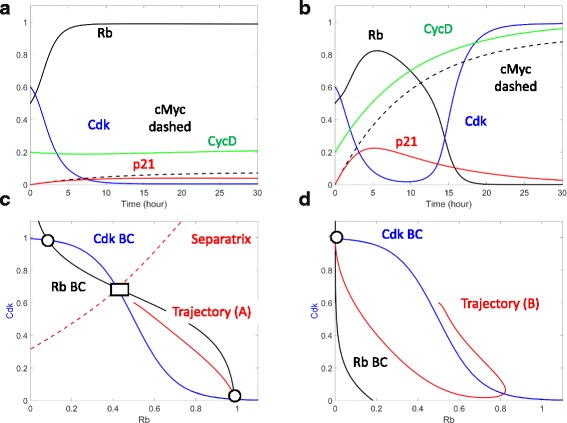
Enhanced cell cycle entry in response to HP infection. **a** and **b** Temporal simulations of Cdk (blue), Rb (black), CycD (green), p21 (red), and c-Myc (black dashed) in the absence of HP (**a**) and in the presence of HP (**b**). **c** Phase plane in the absence of HP infection. The balance curves of Cdk (blue), the balance curves of Rb (black), and separatrix (red) are computed. Also shown are stable attractors (black solid circles) and the unstable saddle point (black solid rectangle). The red temporal trajectory indicates the temporal change of Rb and Cdk from (**a**). **d** Phase plane analysis in the presence of HP infection. The notations are as in (**c**), and the red trajectory is taken from (**b**)

The mitotic signals received by cells set their Rb and Cdk activities. In the absence of mitotic stimulation due to infection, Cdk activity is low (blue solid curve in Fig. 4a) and Rb is active (black solid curve in Fig. 4a). This cell would arrest in the quiescent state. In HP infected cells, the mitotic signal from the bacteria activates the transcription factor cMyc (the black dashed line in Fig. 4b), which then induces the expression of CyclinD and the activation of Cdk (green and blue curves in Fig. 4b). Rb is then inactivated (black curve, Fig. 4b) resulting in active proliferation.

The mutually antagonistic switch between Cdk and Rb controls the decision on the restriction point [19]. By inhibiting the transcription factor E2F and actively recruiting histone deacetylases, Rb represses the transcription of Cyclin A and Cyclin E. In this way, Rb can inhibit Cdk activity because both Cyclins A and E are activating partners of Cdk. This inhibitory regulation is illustrated by the Cdk balance curve (blue curve, Fig. 4c). At low levels of Rb, Cdk activity is high; when Rb activity increases, Cdk activity is brought down. On the other hand, Cdk phosphorylates Rb and represses Rb activity. Hence the Rb balance curve shows high Rb activity only when Cdk activity is low. As Cdk activity increases, Rb is inactivated (black curve, Fig. 4c).

The three intersections between the Rb balance curve and the Cdk balance curve correspond to the three steady states of the restriction point control system. Two of these are stable attractors (black circles, Fig. 4c), and their coexistence has been shown by the response of cells to transient growth factor stimulation [18]. The intermediate intersection (black rectangle, Fig. 4c) corresponds to an unstable steady state. Due to its instability, it cannot be observed experimentally. However it must exist for the two stable attractors to coexist. This is because the unstable steady state serves as the origin of a separatrix (red dashed lines, Fig. 4c). The separatrix is necessary for dividing the plane into two attracting regions: the top, left part is the attracting region of the left attractor; and the bottom, right part is the attracting region of the right attractor.

In the absence of HP, the cell resides in the attracting region of the right attractor. Hence, as time goes by, this cell moves to the right attractor, characterized by high Rb activity and low Cdk2 activity (red dashed trajectory, Fig. 4c). Once reaching this stable attractor, the cell must remain near the attractor and cannot progress through the cell cycle.

Following HP infection, transcriptional activators such as β-catenin are activated [20]. These factors induce cell cycle promoters such as Cyclin D, which inhibit Rb. As Rb is repressed by these cell cycle promoters, less Cdk activity is required to bring down Rb activity. On the phase plane, this event is reflected by the left shift of the Rb balance curve (compare the black curves, Fig. 4c and d). This left movement of the Rb balance curve results in the loss of the right attractor corresponding to a resting cell (with high Rb and low Cdk activity). Consequently, the cell has to move to the left attractor that corresponds to a proliferating cell (characterized by low Rb and high Cdk activity). In this way, HP infection can result in enhanced cell proliferation.

## Discussion

As discussed above, the current model successfully recaptures many experimentally observed cellular and molecular host responses to HP infection. Since the model can successfully mimic the biological system of interest, it serves as an “in silico” representation of the system. The benefit of such a representation is that we can analyze this model with nonlinear dynamical tools and discover deep, mechanistic insights that cannot be directly observed in the biological system.

### Multiscale positive feedbacks might result in a “*ratchet ladder*” and breaks the homeostasis

**Fig. 5:**
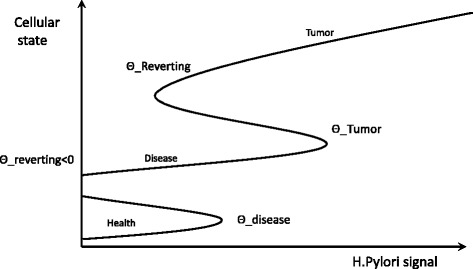
Multiple positive feedbacks contribute to unidirectional disease progression following HP infection. These positive feedbacks form a “Ratchet ladder” that favors the uni-directional progression from heath state to disease state, the implications are elaborated in the discussion

The current model suggests that a chain of multiscale, positive feedbacks, result in a “*ratchet ladder*” in response to the pathological signal from HP (Fig. 5). This mechanistic understanding reveals some significant insights:

1). In healthy cells, these positive feedbacks might provide a “protective threshold”. So long as the signal provided by HP is below this threshold, the cells will not proceed into a diseased state. As a clinical benefit, this protective threshold could be utilized to allow cells to remain in a healthy state.

2). Once healthy cells pass over the tipping point (threshold), they could face extreme difficulty in returning to the initial state. In the most extreme case, the system might stay in the “disease state” even after HP is removed from the system. If true, then even a transient infection by HP might have a permanent effect on patients. Such a sustained pathological consequence after transient HP infection was noticed by Mera et al. [21].

3). This “ratchet ladder” allows for minor alterations (e.g. mutations or inflammation) to accumulate in infected populations. After each minor change, the system moves up one step on the ladder, where it remains. It continuously moves up rungs of the ladder as the system accumulates additional minor changes. This ability to accumulate minor changes promotes the development of gastric disease in such populations and contributes to the pathogenesis of HP infection.

4). In this “ratchet ladder” system, it is easy for the system to move from healthy state to disease state; but it is much more difficult for the system to move from a disease state to a healthy state. Given the protection threshold discussed above, it would be more effective to either prevent infection or to treat it early (before it progresses to more significant pathology).

5). Independent signals can alter the thresholds of these positive feedbacks. Differences in thresholds might then be able to explain the dramatic heterogeneity of HP associated gastric pathology. For example, in populations with enhanced IL-1 production, the required threshold of HP would be reduced, and the rate of gastric tumor increased. This has been shown in a number of populations with IL-1 mutations. Those patients with mutations associated with decreased IL-1 production have been shown to have a protective effect, in that the tumor incident rate in this population is decreased, meaning they have a higher threshold [22].

### The predicted bistable switches are robust features of the model

**Fig. 6:**
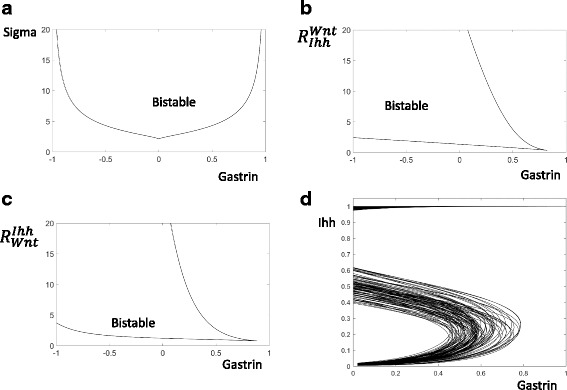
The bistable Ihh switch is robust. **a** and **b** and **c** The bistability area is computed by tracing the Ihh activation and inactivation thresholds (the black curves) when both Gastrin and a second parameters are altered continuously. The names of the second parameters are labelled on y-axis of these panels. **d** The response of 100 different hosts are computed by randomly changing all parameters and computing 100 one bifurcation diagrams

The bistable switches controlling Ihh and Shh are novel predictions of the current model and have not been experimentally tested. In order to examine whether these switches will persist when another parameter changes, we have carried out two parameter bifurcation analysis of these switches. For example, in order to test the robustness of the predicted bistable Ihh switch (Fig. 3d), we use Gastrin as the first parameter, and then trace the positions of the Saddle-node bifurcation upon the change of a second parameter. The bistability region is persistent for a broad ranges of the parameters (Fig. 6a-c), indicating that the predicted bistable switch that controls Ihh activation is a robust feature of the current model. Similarly, the predicted bistable switch controlling Shh activation is also a robust feature of the current model.

### The genetic diversities of the hosts and pathogens will result in diverse responses and signals

Different genetic makeups are expected to characterize different hosts or pathogens, and such genetic diversity is expected to change the shapes of the response curves and the levels of the infecting signals.

In Fig. 6d, we have mimicked the genetic diversity of 100 different hosts by randomly assigning them different values of all the control parameters. As shown by the one parameter bifurcation curves, these 100 hosts are characterized by different thresholds of Ihh activation. On the basis of this, we can expect that different hosts can show diverse responses to identical signals. For a particular level of the signal (e.g. Gastrin = 0.6 in Fig. 6d), Ihh will be activated in some of the hosts but will remain inactive in others.

Different strains of HP can be characterized by differences in expression of the virulence factors, such as CagA and VacA [23]. CagA is expressed in 50% or more of infecting HP. Its presence is commonly associated with severe disease, though there are exceptional instances of severe disease where CagA is absent. Nearly 50% of HP strains secrete the cytotoxin VacA, and the activities of VacA can vary amongst HP strains. During chronic infection, the HP bacterium are able to evolve to acquire features of different strains, and different HP strains can co-exist in the same human host.

Such genetic diversity of the pathogen is expected to result in different levels of the pathogen signal (x-axis of Fig. 5). In addition, the different loading concentrations of pathogen also play a critical role in the model since the HP signal is a function that lumps together the net effect of bacterial concentration as well as virulence. A high concentration of the functional infecting bacterium that express high levels of both CagA and VacA is expected to generate a strong pathogen signal. This strong signal can then result in fast disease progression and more severe symptoms [24]. On the other hand, a low concentration of the bacteria or genetic mutations that weaken the expression of virulence factors is expected to result in a weak pathogen signal and consequently slow disease progression. If different strains of HP coexist in the same host, then the pathogen signal is expected to be a function in the form of Pathogen signal = [load of Strain A] * Virulence of Strain A + [load of Strain B] * Virulence of Strain B + ……..

In this way, the genetic diversity of the hosts and pathogens would likely explain the heterogeneous progression of HP induced disease in a population of infected patients. In a future expansion of the model, it will be crucial to incorporate additional evidence to elaborate the relation between the different genetic composition of hosts, strain load, virulence strength, co-infection of multiple strains and the corresponding disease severities.

### Integrating community efforts to model complex host pathogen interactions

Pathogenesis is an intricate process which involves complex regulations at the pathogen, host, and pathogen-host level. In its current state, our model has not incorporated all the feedbacks that might control the host response to HP infection. For example, the infection leads to an Epithelial-Mesenchymal Transition (EMT) through multiple pathways. Based on our current understanding of EMT [25], the transition is controlled by a tri-stable switch that is defined by the interaction of essential EMT regulators, Snail and ZEB. Due to its complex nature, modeling HP’s host-pathogen interactions is beyond the ability of any single group. Indeed, many groups have made significant contributions to modeling HP infection. Previous models have been developed to investigate pathogen survival mechanisms [26], the interaction between HP and host acid secretion [27, 28], and the host immune response to the infection [7, 29, 30].

The current multiscale, model has been purposely built with various functional modules. Due to this modular nature, the current model can be easily expanded to accommodate existing and new modules of additional interaction pathways. For example, the Shh signaling pathway is characterized by a transcriptional positive feedback in which Gli induces its own expression. In addition, Gli is known to be bifunctional in that they can exist as repressors in the absence of ligand and act as activators if ligand is present [31]. These additional regulations, as well as the EMT switch and the molecular switch that controls programmed cell death, can be incorporated into the model in the future.

An expanded, comprehensive model will help the biomedical community summarize our understanding the HP induced signaling pathways. This summary will prove useful in designing future experiments, studying the dynamics of the involved molecular/cellular processes, examining complex multi-pathway interactions, and predicting novel treatment protocols.

## Methods

The current model describes the complex cellular and molecular responses to HP infection in four modules (Fig. 1).The molecular interaction networks within these modules are constructed on the basis of reported experimental evidence, and these references are provided when the corresponding molecular interactions are described.

### The hedgehog responses module

Shh is a conserved signaling pathway. When Shh ligand is not present, the Shh receptor Patched-1 actively represses Smo and leads to processing of full-length Gliinto its shorter, repressor form. This repressor form of Gli then actively represses the target genes of Shh. In the presence of Shh ligand, Patched-1 no longer represses Smo, leading to the activation of the Gli activator. Gli then activates the transcription of target genes of Shh [31]. For further details on the Shh signaling pathway and its targets we refer readers to an excellent review by Briscoe and Therond [31].

Shh normally promotes the production of SST, as shown in Shh-knocked down mice. After Shh is lost during the gastric atrophy stage, Somatostatins (SST) production is dramatically decreased. This leads to the loss of SST’s inhibitory feedback on gastrin, which in turn results a corresponding increase in gastrin that leads to hypergastrinemia [32].

Gastrin accumulation promotes the production of Ihh in pit cells located in the fundus. Ihh, once produced, activates mesenchymal cells to produce Wnt. The Wnt in turn stimulates the proliferation of pit cells and thus production of even more Ihh. In this way, Indian Hedgehog is activated after the loss of Shh during atrophy [15].

In the late stages of a chronic HP infection, high levels of inflammatory factors (e.g. TGF-β) are maintained. These factors recruit and activate bone marrow (BMMSC). BMMSC produces and secretes Shh, which further enhances the proliferation of BMMSCS in an autocrine fashion [11, 13].

### The module of transcriptional change

Infection with HP leads to the activation of multiple transcriptional factors such as NF-κB [6]. Through the interaction between CagA produced by HP and host CD44, the bacteria is also able to activate the PI3K pathway [17]. PI3K leads to activation of the MAPK/ERK pathway and the activation of transcription factor AP1. In addition, PI3K leads to the inactivation of Gsk3-beta, leading to stabilization and nuclear accumulation of β-catenin [20]. Β-catenin then promotes the transcription of downstream genes. The Wnt/ β-catenin pathway contains its own signaling networks and regulations which are not included in the mode. For further details about this pathway and its target genes, we refer readers to an excellent review by Macdonald et al. (Wnt) [33]. These transcriptional changes normally occur in the stomach antrum.

### The atrophy module

The stomach is a highly acidic environment, with a resting pH between 1.5 and 3.5. This acidic environment is maintained by parietal cell H-K ATPases. Acid secreted by these cells activates pepsin which aids in the activation of Shh. In addition, acid activates Protein Kinase C and promotes the transcription of Shh. In return, Shh promotes the transcription of the HK-Atpase [34]. The observed atrophy occurs in the fundus.

### The restriction point module

The restriction point in mammalian cells is controlled by the mutual antagonism between Rb and Cdk. By repressing E2F activity and recruiting histone deacetylase, Rb repressing the transcription of cyclins A and E. Since these cyclins are essential activating partners of Cdk, Rb effectively represses Cdk activity. In return, Cdk phosphorylate Rb, and such phosphorylation converts Rb into its inactive form.

Through the activation of transcription factors AP1 and β-catenin, HP is able to promote the transcription of cyclin D. Cyclin D binds to Cdk4/6, and thus activated Cdk 4/6 phosphorylates and inhibits Rb [35].

In polarized cells, HP interacts with PAR1 (partitioning defective 1) through its pathogenic protein CagA. The CagA:PAR1 complex promotes the membrane localization and activation of GEF-H1 (Guanine nucleotide exchange factor H1). Activated GEF-H1 in turn activates RhoA and RhoA associated kinase. This RhoA signaling pathway eventually activates the transcription factor c-Myc. Activated c-Myc induces multiple micro RNAs and prevents the accumulation of p21. In non-polarized cells, GEF-H1 is localized in the cytoplasm and cannot be activated by HP. Due to insufficient activity of c-Myc, p21 is accumulated in these cells [36]. The AP-1 transcription factor as well as it targets may contain additional regulations that are not discussed here we refer readers to an excellent review by Shaulian et al. [37] for additional details regarding this pathway.

### Model construction

All model components were summarized into the wiring diagram presented in Fig. 1. Once summarized, the diagram was converted to a system of ordinary differential equations using a generic formula as previously described [35, 38, 39]. For more efficient computation, linear pathways were summarized and only the input/output relationships were taken into account.

Though the structure of the control network is well supported experimentally, the values of the parameters characterizing these network reactions are mostly unknown and have to be estimated as part of the model construction process. The values of these parameters were estimated using a “*trial and error*” approach where upon each parameter was altered till resulting simulations matched literature reported experimental results. The model equations and the estimated values of the model parameters are presented in Additional file 1: Table S1. A summary of the model simulations and the corresponding experimental observations is presented in Table 1.

**Table 1:** Model simulation and corresponding experimental evidence

Model simulation	Experimental evidence
IkB loss and Recovery (Fig. 2a)	Figures 4a & b, Schumacher et al., 2015, [6]
IL-1 Increase and Shh/Acid Decrease (Fig. 2b)	Figures 5a & 5b, Waghray et al. 2009, [10]
Recovery and BMMSC/TGF-B Increase (Fig. 2c)	Figure 1, Houghton et al. 2004, [11]; Fig. 3, Varon et al. 2012, [12]
Gastrin, Wnt, Ihh Increases (Fig. 3c)	Figures 3, 5, 6 Feng et al. 2014, [15]

### Dynamical analysis

Time series simulations and phase plane analyses were performed using XppAut ((http://www.math.pitt.edu/~bard/xpp/xpp.html). Bifurcation analysis was done using Oscill8 (http://oscill8.sourceforge.net/). All figures were plotted in MATLAB (https://mathworks.com/).

## Additional file

Additional file 1: Table S1. Model equations and parameters. (DOCX 32 kb)
