# Supplementary material for: Multiscale positive feedbacks contribute to unidirectional gastric disease progression induced by helicobacter pylori infection
Source: BMC Syst Biol. 2017 Nov 22;11:111. doi: 10.1186/s12918-017-0497-y (PMC5700561; doi:10.1186/s12918-017-0497-y)
Supplement: Additional file 1: Table S1. — Model equations and parameters. (DOCX 32 kb) [file 12918_2017_497_MOESM1_ESM.docx]

| **Restriction Point Module** | | | | | |
| --- | --- | --- | --- | --- | --- |
| **cMyc** | | | | | |
| $w_{Myc}=R_{0}^{Myc}+R_{HP}^{Myc}\cdot HP$ | | $F_{Myc}=\frac{1}{1+e^{-\sigma\cdot wMyc}}$ | | $\frac{dMyc}{dt}=ts_{Myc}\cdot(F_{Myc}-Myc)$ | |
| **cMyc parameters** | | | | | |
| $R_{0}^{Myc}= -0.5$ | | $R_{HP}^{Myc}=1$ | | $ts_{Myc}=0.1$ | |
| **P21** | | | | | |
| $w_{p21}=R_{0}^{p21}+R_{HP}^{p21}\cdot HP+R_{Myc}^{p21}\cdot Myc$ | | $F_{p21}= \frac{1}{1+ e^{-\sigma\cdot w_{p21}}}$ | | $\frac{dp21}{dt}=ts_{p21}\cdot(F_{p21}-p21)$ | |
| **P21 parameters** | | | | | |
| $R_{0}^{p21}= -0.5$ | $R_{HP}^{p21}=1$ | | $R_{Myc}^{p21}= -2$ | | $ts_{p21}=0.1$ |
| **CyclinD** | | | | | |
| $w_{CycD}=R_{0}^{CycD}+R_{HP}^{CycD}\cdot HP+R_{Myc}^{CycD}\cdot Myc$ | | $F_{CycD}= \frac{1}{1+e^{-\sigma\cdot w_{CycD}}}$ | | $\frac{dCycD}{dt}=ts_{CycD}\cdot(F_{CycD}-CycD)$ | |
| **CyclinD parameters** | | | | | |
| $R_{0}^{CycD}= -0.33$ | $R_{HP}^{CycD}=1$ | | $R_{Myc}^{CycD}=1$ | | $ts_{CycD}=0.1$ |
| **RB** | | | | | |
| $w_{Rb}=R_{0}^{Rb}+R_{Cdk}^{Rb}\cdot Cdk2+R_{CycD}^{Rb}\cdot CycD$ | | $F_{Rb}= \frac{1}{1+e^{-\sigma\cdot w_{Rb}}}$ | | $\frac{dRb}{dt}=ts_{Rb}\cdot(F_{Rb}-Rb)$ | |
| **RB parameters** | | | | | |
| $R_{0}^{Rb}=1.2$ | $R_{Cdk}^{Rb}= -1.4$ | | $ts_{Rb}=1$ | | $R_{CycD}^{Rb}= -1.5$ |
| **CdK** | | | | | |
| $w_{Cdk}=R_{0}^{Cdk}+R_{Rb}^{Cdk}\cdot Rb+R_{p21}^{Cdk}\cdot p21$ | | $F_{Cdk}= \frac{1}{1+e^{-\sigma\cdot w_{Cdk}}}$ | | $\frac{dCdk2}{dt}=ts_{Cdk}\cdot(F_{Cdk}-Cdk2)$ | |
| **CdK parameters** | | | | | |
| $R_{0}^{Cdk}= 1$ | $R_{Rb}^{Cdk}= -2$ | | $R_{p21}^{Cdk}= -2$ | | $ts_{Cdk}=0.5$ |
| **Global Parameters** | | | | | |
| $\sigma=5$ | | | $HP=1$ | | |

| **Transcriptional Response Module** | | | | | |
| --- | --- | --- | --- | --- | --- |
| **NfKb** | | | | | |
| $W_{NFkB}=R_{0}^{NFkB}+R_{IkB}^{NF}\cdot IkB$ | | $F_{NFkB}= \frac{1}{1+e^{-\sigma\cdot W_{NFkB}}}$ | | $\frac{dNFkB}{dt}=ts_{NFkB}\cdot(F_{NFkB}-NFkB)$ | |
| **NfKb parameters** | | | | | |
| $R_{0}^{NFkB}=0.4$ | $R_{IkB}^{NF}= -1.2$ | | $ts_{NFkB}=2$ | | $\sigma=5$ |
| **ImR** | | | | | |
| $W_{ImR}=R_{0}^{ImR}+R_{NFkB}^{ImR}\cdot NFkB$ | | $F_{ImR}= \frac{1}{1+e^{-\sigma\cdot W_{ImR}}}$ | | $\frac{dImR}{dt}=ts_{ImR}*(F_{ImR}-ImR)$ | |
| **ImR parameters** | | | | | |
| $R_{0}^{ImR}=-0.4$ | | $R_{NFkB}^{ImR}=1.5$ | | $ts_{ImR}=0.1$ | |
| **IKB** | | | | | |
| $W_{IkB}=R_{0}^{IkB}+R_{HP}^{IkB}\cdot HP+R_{ImR}^{IkB}\cdot ImR$ | | $F_{IkB}= \frac{1}{1+e^{-\sigma\cdot W_{IkB}}}$ | | $\frac{dIkB}{dt}=ts_{IkB}\cdot(F_{IkB}-IkB)$ | |
| **IKB parameters** | | | | | |
| $R_{0}^{IkB}=0.3$ | $R_{HP}^{IkB}= -1$ | | $R_{ImR}^{IkB}=2.4$ | | $ts_{IkB}=2$ |
| **NfKB- Downstream Genes** | | | | | |
| $W_{IL8}=R_{0}^{IL8}+R_{NFkB}^{IL8}\cdot NFkB$ | | $F_{IL8}= \frac{1}{1+e^{-\sigma\cdot W_{IL8}}}$ | | $\frac{dIL8}{dt}=ts_{IL8}\cdot(F_{IL8}-IL8)$ | |
| **Downstream Gene Parameters** | | | | | |
| $R_{0}^{IL8}=-0.4$ | | $R_{NFkB}^{IL8}=1.5$ | | $ts_{IL8}=1$ | |
| **Beta_Catenin** | | | | | |
| $W_{Bet}=R_{0}^{Bet}+R_{HP}^{Bet}\cdot HP$ | | $F_{Bet}=\frac{1}{1+e^{-\sigma\cdot W_{Bet}}}$ | | $\frac{dBet}{dt}=ts_{Bet}\cdot(F_{Bet}-Bet)$ | |
| **Beta-Catenin Parameters** | | | | | |
| $R_{0}^{Bet}=-0.4$ | | $R_{HP}^{Bet}=1$ | | $ts_{Bet}=0.2$ | |

| **HH Response Module** | | | | | | | | | | | | | | | | | | | | | | | | | | |
| --- | --- | --- | --- | --- | --- | --- | --- | --- | --- | --- | --- | --- | --- | --- | --- | --- | --- | --- | --- | --- | --- | --- | --- | --- | --- | --- |
| **Wnt Equations** | | | | | | | | | | | | | | | | | | | | | | | | | | |
| $W_{Wnt}=R_{0}^{Wnt}+ R_{Gas}^{Wnt} \cdot Gas+ R_{Ihh}^{Wnt} \cdot Ihh$ | | | | | $F_{Wnt}=\frac{1}{1+ e^{-\sigma\cdot W_{Wnt}}}$ | | | | | | | | | | | | $\frac{dWnt}{dt}= {ts}_{Wnt}\cdot(F_{Wnt}-Wnt)$ | | | | | | | | | |
| Wnt Parameters | | | | | | | | | | | | | | | | | | | | | | | | | | |
| $R_{0}^{Wnt}$ = -1 | | | | | $R_{Ihh}^{Wnt}= 2$ | | | | | | | | $R_{Gas}^{Wnt}=1$ | | | | | | | | | | ${ts}_{Wnt}=1$ | | | |
| **Ihh Equations** | | | | | | | | | | | | | | | | | | | | | | | | | | |
| $W_{Ihh}=R_{0}^{Ihh}+ R_{Wnt}^{Ihh} \cdot Wnt$ | | | | | $F_{Ihh}=\frac{1}{1+ e^{-\sigma\cdot W_{Ihh}}}$ | | | | | | | | | | | | | | $\frac{dIhh}{dt}= {ts}_{Ihh}\cdot(F_{Ihh}-Ihh)$ | | | | | | | |
| Ihh Parameters | | | | | | | | | | | | | | | | | | | | | | | | | | |
| $R_{0}^{Ihh}$ = - 0.9 | | | | $R_{Wnt}^{Ihh}$ = -1 | | | | | | | | | | | | | ${ts}_{Ihh}=1$ | | | | | | | | | |
| **Shh Equations** | | | | | | | | | | | | | | | | | | | | | | | | | | |
| $W_{\mathrm{Shh}}= R_{0}^{Shh}+ {(R}_{Acid}^{Shh}\cdot Acid)+ {(R}_{BMMSC}^{Shh}\cdot BMMSC)+{(R}_{IL1}^{Shh} \cdot IL1)$ | | | | | | | | | | | | | | $F_{Acid}= \frac{1}{1+ e^{-\sigma\cdot W_{Shh}}}$ | | | | | | | | | $\frac{dShh}{dt}= {ts}_{Shh}\cdot(F_{Shh}-Shh)$ | | | |
| Shh Parameters | | | | | | | | | | | | | | | | | | | | | | | | | | |
| $R_{0}^{Shh}= -0.9$ | $R_{Acid}^{Shh}=1.8$ | | | | | | | $R_{BMMSC}^{Shh}$ = 2.3 | | | | | | | $R_{IL1}^{Shh}= -1$ | | | | | | | | | | | ${ts}_{Shh}=1$ |
| **TGF Equations** | | | | | | | | | | | | | | | | | | | | | | | | | | |
| ${W_{TGF}= R}_{0}^{TGF}+ R_{HP}^{TGF}\cdot HP$ | | | | | $F_{TGF}= \frac{1}{1+ e^{-\sigma\cdot W_{TGF}}}$ | | | | | | | | | | | | | | | | $\frac{dTGF}{dt}= {ts}_{TGF}\cdot(F_{TGF}-TGF)$ | | | | | |
| TGF Parameters | | | | | | | | | | | | | | | | | | | | | | | | | | |
| $R_{0}^{TGF}= -0.5$ | | | | | | | $R_{HP}^{TGF}=1$ | | | | | | | | | | | | | | | | | ${ts}_{TGF}=0.005$ | | |
| **BMMSC Equations** | | | | | | | | | | | | | | | | | | | | | | | | | | |
| ${W_{BMMSC}= R}_{0}^{BMMSC}+ R_{TGF}^{BMMSC}\cdot TGF+ R_{Shh}^{BMMSC}\cdot Shh$ | | | | | | | | | $F_{BMMSC}= \frac{1}{1+e^{-\sigma\cdot W_{BMMSC}}}$ | | | | | | | | | | | $\frac{dBMMSC}{dt}= {ts}_{BMMSC}\cdot(F_{BMMSC}-BMMSC)$ | | | | | | |
| BMMSC Parameters | | | | | | | | | | | | | | | | | | | | | | | | | | |
| $R_{0}^{BMMSC}= -1.5$ | | | $R_{Shh}^{BMMSC}=1$ | | | | | | | | | $R_{TGF}^{BMMSC}=5$ | | | | | | | | | | | | | ${ts}_{BMMSC}=0.01$ | |
| **Gas Equations** | | | | | | | | | | | | | | | | | | | | | | | | | | |
| ${W_{Gas}= R}_{0}^{Gas}+ R_{HP}^{Gas}\cdot Shh$ | | | | | | | | $F_{Gas}= \frac{1}{1+ e^{-\sigma\cdot W_{Gas}}}$ | | | | | | | | $\frac{dGas}{dt}= {ts}_{Gas}\cdot(F_{Gas}-Gas)$ | | | | | | | | | | |
| Gas Parameters | | | | | | | | | | | | | | | | | | | | | | | | | | |
| $R_{0}^{Gas}=0.5$ | | | | | | $R_{HP}^{Gas}= -1$ | | | | | | | | | | | | | | | | | | ${ts}_{Gas}=0.01$ | | |
| **Atrophy Module** | | | | | | | | | | | | | | | | | | | | | | | | | | |
| **IL-1 Equations** | | | | | | | | | | | | | | | | | | | | | | | | | | |
| ${W_{IL1}= R}_{0}^{IL1}+ R_{HP}^{IL1}\cdot HP$ | | | | $F_{IL1}= \frac{1}{1+ e^{-\sigma\cdot W_{IL1}}}$ | | | | | | | | | | | | | | $\frac{dIL1}{dt}= {ts}_{IL1}\cdot(F_{IL1}-IL1)$ | | | | | | | | |
| IL-1 Parameters | | | | | | | | | | | | | | | | | | | | | | | | | | |
| $R_{0}^{IL1}= -1$ | | | | | $R_{HP}^{IL1}=1$ | | | | | | | | | | | | ${ts}_{IL1}=2$ | | | | | | | | | |
| **Acid Equations** | | | | | | | | | | | | | | | | | | | | | | | | | | |
| $W_{\mathrm{Acid}}= R_{0}^{Acid}+ {(R}_{IL1}^{Acid}\cdot IL1)+ {(R}_{Shh}^{Acid}\cdot Shh)$ | | | | | | | | | | $F_{Acid}= \frac{1}{1+e^{-\sigma\cdot W_{Acid}}}$ | | | | | | | | | | | | $\frac{dAcid}{dt}= {ts}_{Acid}\cdot(F_{Acid}-Acid)$ | | | | |
| Acid Parameters | | | | | | | | | | | | | | | | | | | | | | | | | | |
| $R_{0}^{Acid}= -1$ | | $R_{Shh}^{Acid}=2$ | | | | | | | | | $R_{IL1}^{Acid}= -1.8$ | | | | | | | | | | | ${ts}_{Acid}=1$ | | | | |
